# Psychometric properties of Nepalese preschool anxiety scale among preschool children: A cross‐sectional study

**DOI:** 10.1002/hsr2.808

**Published:** 2022-09-03

**Authors:** Sabina Maharjan, Mita Rana, Bidusha Neupane, Sujan Rijal, Suraj Shakya, Pramesh Man Pradhan, Saroj Prasad Ojha, Kamal Gautam, Rakesh Singh

**Affiliations:** ^1^ Transcultural Psychosocial Organization Nepal Kathmandu Nepal; ^2^ Department of Psychiatry and Mental Health, Institute of Medicine Tribhuvan University Kathmandu Nepal; ^3^ School of Health Service Old Dominion University Norfolk Virginia USA; ^4^ Health Economics Policy and Management University of Oslo Oslo Norway

**Keywords:** anxiety, confirmatory factor analysis, preschool anxiety

## Abstract

**Background:**

The Preschool Anxiety Scale (PAS)‐Parent version scale is a 28‐item measure designed to assess anxiety symptoms in preschoolers aged 3−6 years. The aim of this study was to assess the psychometric properties of the Nepali translated version of the PAS‐Parent version.

**Methods:**

A descriptive cross‐sectional design was used to collect data from 680 mothers among seven conveniently selected schools in Kathmandu.

**Results:**

The difference in PAS‐Parent version scores across age groups was found to be statistically significant. In confirmatory factor analysis, 28 items showed a poor fit of the five‐factor original model for the data. However, removing three items (25 item version) through the five‐factor model indicated a better fit. Internal consistency measured by Cronbach's *α* for the PAS‐Parent version scale was of good range (0.87). Cronbach's *α* of the subscales: generalized anxiety (0.63), social phobia (0.67), physical injury fears (0.75), and separation anxiety (0.63) were in fair range; while it was in poor range for the obsessive‐compulsive subscale (0.567).

**Conclusion:**

Nepali version of the PAS demonstrated fair psychometric properties, supporting its utility in screening and assessing a broad range of anxiety symptoms in Nepalese preschoolers.

## 1 INTRODUCTION

Anxiety is a normal part of childhood and every child goes through phases that can be characterized by persistent, irrational, overwhelming worries, fear, and anxiety that interfere with daily activities. Globally, 10%−20% of children and adolescents suffer from mental disorder, with half it beginning by the age of 14, and three‐quarters before the age of 25.^1^, ^2^ In Nepal, 40% of the population are younger than 18 years of age, and a large proportion of the population that is at risk of developing a mental disorder.^3^, ^4^ The clinical prevalence of anxiety disorders was reported ranging from 18.8% to 24.4% while that of Attention Deficit Hyperactivity Disorder was 10%–11.7% in various clinical samples of children and adolescents in Nepal.^5^

Anxiety disorders are the most common class of mental disorders across development.^6^, ^7^ and the median age of onset was found to be 6 years for affected youth.^8^ Hence this disorder begins very early in life with the prevalence around 9% in preschool population.^9^

Anxiety disorder studies have shown concerning stability over preschool period indicating that 34% of children with an anxiety disorder at age 3 continue to meet criteria for diagnosis until age 6.^10^ In longer‐term, childhood anxiety disorder often follows a chronic or recurring course into adolescence and adulthood.^11^ In addition, the prevalence of anxiety disorders in preschool‐aged children is similar to children aged 5−17 years.^9^ Likewise, anxiety disorder may also predict externalizing and internalizing childhood disorders such as depression and conduct disorder which may occur later in life.^11^

Anxiety is another key characteristic of the behaviorally inhibited child. Anxiety disorders in children are common and affects about 5%−17% of children.^12^ If the behavioral inhibition is long been implicated in development of internalizing problem children experience anxiety related somatic symptoms, stomach pain, sleeplessness, enuresis, constipation, allergies, and asthma.^13^

This behavioral inhibition will lead to shyness and social withdrawal in later life. The characteristic manifests differently in different development ages: inhibited toddlers react to novelty with agitation, distress as well as clinging to the caregiver; preschoolers react with hesitancy, inhibited spontaneous conversation, and school children manifest inhibition through extreme shyness and constriction with unfamiliar adults, and through quiet isolation with unfamiliar peers. Subsequently in adolescents frequently express behavioral inhibition through social withdrawal, social phobia, and in some instances aggression and violence.^13^ This leads to loss in productivity, school absenteeism and more functional impairment as child grow older. Hence, ability to detect psychiatric disorder early is important.^14^

There is a lack of study and human resources working specifically on mental health and anxiety among preschoolers in Nepal. One of the barriers to research into preschool anxiety is the lack of reliable and valid assessment tools. There are relatively little research about anxiety problem among preschooler.^15^ The importance of developing adequate strategies for assessing and treating anxiety disorders in preschool‐aged children is important to carry out.

The Preschool Anxiety Scale (PAS) is the only measure that specifically assesses multiple anxiety symptoms in preschool‐aged children.^16^ The PAS is 28‐item parent report measure that was designed to assess anxiety dimension specified in Diagnostic and Statistical Manual of Mental Disorders‐IV.^16^ The factor structure and construct validity of PAS were examined in a large Australian community sample.^16^ Confirmatory factor analysis showed five factors: separation anxiety disorder, physical injury fears (PIFs), social phobia, obsessive‐compulsive disorder and generalized anxiety disorder (GAD). Anxiety scores were generally higher than the ones reported by Spence.^17^ Symptoms of PIFs and social anxiety were the most common, but had found limited evidence for gender or age differences. The PAS scale shows good reliability and construct validity in community scale of children 3−5 years. In other countries such as Chinese,^17^ Dutch,^18^ Romanian,^19^ Spanish^20^ children, being the original five‐factor structure proposed by Spence^16^ shows good psychometric properties of PAS. A different structure with a five factor model that excluded separation anxiety factor and representing PIF has been found in a Dutch sample.^18^ Likewise, in Spanish sample while conducting confirmatory factor analysis (CFA) of five factor model original model, 8 items were eliminated because of their low correlation item scale such as separation anxiety (3 items), social anxiety (4 items) and generalized anxiety (1 items).^21^

The purpose of this study was to test the psychometric properties of the Spence PASs.^16^ Therefore, this study intended to examine the psychometric properties of the Nepali translated version of PAS‐Parent version in private schools of Kathmandu. Internal consistency of the total scale and subscale was assessed.

## 2 MATERIALS AND METHODS

### 2.1 Study context

The current population of Nepal is 29,192,480 as per the 2021 census. The population growth rate is 0.93% per year. In the 2011 census, Nepal's population was approximately 26 million people with a population growth rate of 1.35% and a median age of (21.6 years). The demographic statistics castes/ethnics group of Nepal is Chhetri (16.6%), Brahmin (12.2%), Newar (5.9%), Tamang (5%), Muslim (4.4%), and others (44.1%).^20^ According to 2011 census revealed that 81.3% of Nepal population was Hindu, 9.0% Buddhist, 4.4% Muslim, 3% Kiratist, 1.42% Christian and 0.9% followed other or no religion.^20^ Kathmandu is capital city of Nepal. Most of facilities are available in Kathmandu so most of people reside from 75 districts and there is found of mixed communities. So researcher choose this Kathmandu for further study which is relevant also.

### 2.2 Study participants

The sample consisted of 680 children (379 boys and 301 girls) aged between 3 and 6 years. The age distribution was as follows: 15.29% (*n* = 104) were 3 years old; 22.2% (*n* = 151) were 4 years old; 27.79% (*n* = 189) were 5 years old and 34.70% (*n* = 236) were 6 years old. For collecting data, the assessment instrument was completed by their mother (*n* = 680). They were recruited from six schools in Kathmandu valley of Nepal.

### 2.3 Sampling method

The schools were selected using convenient sampling method. Most of the participants were in age groups of 26−30 years (36.3%) where as the least number of participants (0.3%) were representing the age group of (15−20) years. About, 27.9% of the mothers reported that they were literate without formal education and only 8.6% had education till master level. The majority of the participants were housewives (45.2%) and the least number of participants (3.7%) held government jobs. The majority of participants were from joint families (52.9%). Only 7.79% of the mothers reported having a history of mental illness in their family.

### 2.4 Sample size

The sample size has been determined as per the requirement for factor analysis. Several authors have mentioned about criteria of samples in relation to number of items in the questionnaire. For instance, 3:1, 6:1, 10:1, 15:1, or 20:1.^22^, ^23^, ^24^ For current study, 20:1 was taken as per rule of thumb; therefore, as there were 34 questions in the PAS the minimum sample size was 680 in this study. Thus, the required mother's sample was 680 for confirmatory factor analysis. To reach this sample size, 800 participants were approached among which 120 did not agree to participate in the study leading to the response rate of 85% in this study.

### 2.5 Measures

#### 2.5.1 PAS

The PAS originally is comprised of 34 items. During assessment, among the preschool children the mothers reported that there was no any PTSD symptoms so we removed 6 items from PAS. Likewise, the PTSD symptoms were not include in factor analysis because there is no occurrence of traumatic events in preschooler.^4^

The PAS is comprised of 28 items providing information about anxiety and worries in children from 3 to 6 years. The PAS‐parent version consists of five subscales: social anxiety disorder, PIFs, social phobia, obsessive‐compulsive disorder, and GAD, post‐traumatic stress disorder. The participants were asked to rate the items of each subscale on a 5‐point scale ranging from 0 (not true at all) to 4 (very often true). Construct validity of the scale was good.^16^ The permission to utilize the tool was obtained from the lead author.^16^

The PAS was translated into Nepali by a bilingual, mental health expert in psychology, teacher and profession by clinical background. The translated version of the tool (Nepali version) was reviewed by three clinical psychologists from Tribhuvan University Teaching (TUTH). It was followed by back translation of the Nepali tool into English by a bilingual anthropology expert. To study the discrepancies, the two English copies were compared by the researchers and two mental health experts and found that there was no discrepancy in the content and meaning of the items of the tool; hence, the Nepali version of the tool was found to be equivalent and relevant to be pretested among the study participants.

The translated Nepali version of the tool was pretested among 10 mothers of preschool children through self‐administration in presence of the first author. The education background of the mother was from intermediate, bachelor. The participated mothers in the pretesting of the tool had opportunity to ask with the researcher the meaning of items that were not clear to them. The pretesting process indicated that the mothers could easily acquaint with the translated tool of PAS (Nepali version). Nepalese and English version of the adapted tool is presented as supplementary files (Supporting Information: File 1 and Supporting Information: File 2). Moreover, it was found that the questions were easily understood by the respondents and required no modification in the adapted questionnaire. This pretested Nepali version of PAS was used to determine its psychometric properties through cross‐sectional survey among 680 mothers of preschool children in Kathmandu, Nepal.

### 2.6 Study procedure

Approval was taken from Institute Review Board, Maharajgunj Medical Campus, TUTH. The permission was taken from the administrative level and teachers of selected different schools. Preschooler children were considered eligible for the study if they were 3−6 years old and their mother who were willing to participate. A sealed envelope containing a request letter to parents, an informed consent form, and Nepali version of the PAS were sent to parents through the school authority. The questionnaires returned by parents were collected from the school administration.

### 2.7 Data analysis

Analyses were carried out using Statistical Package for Social Sciences (SPSS v.20). Confirmatory factor structure was conducted with SPSS Analysis of Moment Structures 21.0 for five correlated factors original model of PAS provide a good fit of data for the Nepalese sample by the means of the following criteria: *χ* ^2^ equivalent in Confirmatory Factor Analysis (CMIN/DF) with a value equal to 5,^25^ Goodness of Fit Index value is greater than 0.80^26^; Normed Fit Index values is greater than 0.90^25^; Comparative Fit Index value is greater than 0.90^24^; Root Mean Square Error of Approximation value is less than 0.06,^25^ Root Mean Square Residual value is less than 0.06.^26^

## 3 RESULTS

The finding showed that total PAS (mother) score was significantly associated with mother education (*p* < 0.007), mothers' occupation (*p* < 0.001), family types (*p* < 0.003), Caste (*p* < 0.004). Similarly, the total PAS score (Preschool children) significantly associated with age of children (*p* < 0.033) and sex (*p* < 0.01).

### 3.1 Confirmatory factor analysis

Model 1 (Five factors original model): The five models GAD, Social Anxiety Disorder, Separation Anxiety Disorder (SA), PIF, and obsessive‐compulsive disorder (OC) proposed by Spence^16^ was tested allowing correlations between factors. The model provided a poor fit for the data. CFA using the original factor model revealed that 25 of the 28 items had loading in excess of 0.40 on the single factor, and another three items had a loading 0.30. The three items are: Item 3: Related to Obsession and compulsion—(Keeps checking that he/she has done things right) (e.g., that he/she closed a door, turned off a tap) Item 2: Related to Social Phobia—(Worries that he/she will do something to look stupid in front of other people) Item 22: Related to Separation anxiety: (Is reluctant to go to sleep without you or to sleep away from home) Model 2 (Adjusted model): The original five factors models were adjusted by removing three items with Standardized Regression Weights less than 0.4. After this adjustment, this model showed better fit for the data as indicated by the indices in Table 1.

**Table 1:** Confirmatory factor analysis of PAS (mother)

Indices	Model 1	Model 2	Cut off values
*χ* ^2^	1244.806	783.4	‐
Df	342	260	‐
*p*	<0.000	<0.000	≤0.05
CFI	0.798	0.86	>0.90
NFI	0.743	0.568	>0.90
RMR	0.102	0.85	<0.06
RMSEA	0.06	0.054	<0.06
CMIN/DF	3.64	3	5.0
GFI	0.875	0.91	>0.80

Abbreviation: CFA, confirmatory factor analysis; CFI, Comparative Fit Index; CMIN/DF, discrepancy divided by degree of freedom; DSM, Diagnostic and Statistical Manual of Mental Disorders; GFI, Goodness of Fit Index; NFI, Normed Fit Index; OC, obsessive‐compulsive disorder; RMR, Root Mean Square Residual; RMSEA, Root Mean Square Error of Approximation, SA, separation anxiety disorder; TUTH, Tribhuvan University Teaching Hospital.

### 3.2 Reliability analysis of test instrument

The internal consistency (α) of PAS‐Parent version of original scale (28 item version) and adjusted model (25 item version) in Nepalese context was 0.881 and 0.875 respectively which indicate the good range in Table 2. In the original model, the internal consistency of the subscales ranged from poor to fair level: 0.65 for generalized anxiety; 0.67 for social phobia; 0.55 for obsessive‐compulsive disorder; 0.69 for PIF, and 0.62 for separation anxiety.

**Table 2:** Internal consistency of PAS‐Parent version

	Original scale (28 items)		Adjusted model (25 items)	
	Number of items	Cronbach ***α***	Number of items	Cronbach ***α***
Generalized anxiety	5	0.65	5	0.63
Social phobia	6	0.67	5	0.66
Obsessive‐compulsive	5	0.55	4	0.56
Physical injury fears	7	0.69	7	0.75
Separation anxiety	5	0.62	4	0.63
Total score	28	0.88	25	0.87

In the modified model, internal consistency of the subscales ranged from poor to fair level: 0.63 for generalized anxiety; 0.66 for social phobia; 0.56 for obsessive‐compulsive disorder (0.56); 0.75 for PIFs; and 0.63 for separation anxiety.

## 4 DISCUSSION

The objective of the study was to examine the factorial structure and the psychometric properties of the PAS in a Nepalese sample of preschool‐aged children between 3 and 6 years old. The majority of the participants were Hindu mothers between the age ranges of 26−30 years. They were mostly Brahmin and housewives. The majority of them were from joint families. The children were between the age group of 3−6 years. The difference in PAS scores across age groups was found to be statistically significant. Thus, the elevated level of anxiety symptoms in older children may be a response to the transition to kindergarten or school. As the child gets older the scores were higher on PAS. Hence there might be the need for separate age‐specific norms or cut‐off values for PAS scores. Similarly, findings have been reported in Australia and China, where the younger children displayed higher anxiety levels than older children.^16^, ^17^ In context of Nepal, most of child were more attached to their parents and caregiver such as caring in their rearing of child and spend more time together with family member. This could be also the preschool child just enter into kindergarten and started new period in their life at age of 3−4 years. They may not only suffer from fear of leaving their parents but also experience difficulties adjusting to kindergarten with new environment and teachers. Thus, the elevated level of anxiety symptoms in younger children may be a response to this transition stage of their life event. The high level of anxiety symptoms in younger children may be related to their life transition.^17^ Maternal anxieties were also associated with child anxiety. Similarly, insecure attachment and behavioral inhibition can be associated with child anxiety. Likewise, the highest levels of anxiety were shown by children who were behaviorally inhibited and insecurely attached and whose mothers were also anxious.^27^ Children with secure attachments from parents and teachers showed higher reaction time and better auditory, visual, and visual spatial selectivity and maintenance.^28^

The score of PAS was found to differ significantly across sex in this study. However, some studies^29^ revealed PAS score to have no significant difference across sex of preschool children. In context of Nepal sex is strong determinant of school participation. Furthermore, education inequality is based on their gender social inequality in Nepal. Girls are more likely to get education from government school where as boys got education from the private school. Young girls are more likely to obtain less years of schooling than boys because their parents thought their children's preparation for their adult marital roles differently based on gender. Women are expected to leave their families household for their husbands after marriage. Hence, there is more chance of school dropout due to more household activities, no proper education and gender role. This prejudice from family member may have led to increase children anxiety level in gender also.^17^, ^30^

The confirmatory factor analyses suggested that PAS 28 items reflect the dimensions of social phobia, separation anxiety, obsessive‐compulsive disorder, fears of PIF, and generalized anxiety, provide a poor fit of the data. However, the five factors correlated model was found to be a good fit in different studies conducted in Austrian, Romanian and Chinese.^16^, ^17^ Confirmatory Factor Analysis of five‐factor models was adjusted by removing three items with Standardized Regression Weights less than 0.4. After this removal, the new model PAS‐25 shows a better fit for the data on the based‐on mother report. The three items were: Keeps checking that he/she has done things right (obsessive‐compulsive subscale), Worries that he/she will do something to look stupid in front of other people (social phobia subscale), Is reluctant to go to sleep without you or to sleep away from home (separation anxiety subscale). The items on keeping checking things for doing correctly have also been reported to be rare among Australian preschool children^16^ and Chinese preschoolers.^17^ Regarding the items on worry about looking stupid in front of other people and reluctance to sleep without parents, there could be diverse meanings in our culture; and hence there might have been difficulty in comprehension.^31^ For instance, children in our society are allowed to sleep with their parents most of the time. And hence the question on reluctance to sleep without parents could be giving different meaning for Nepali mothers. Similarly, items on is reluctant to go sleep without you or to sleep away from home have been reported in Spanish preschool children.^21^ In current study, Cronbach's alpha for 28 items PAS was 0.88 indicating good reliability. Cronbach's *α* of the generalized anxiety (0.65), Social phobia (0.675), PIFs (0.695), and Separation anxiety (0.627) were in fair range. Obsessive‐compulsive disorder has poor internal consistency, (0.56). The internal consistency of PAS in preschool children of China, Netherland and Portugal reported in similar range.^17^ After removing three items, Cronbach's *α* coefficient for the PAS of 25 items in Nepalese context was 0.87 which was still in good range. Similarly, after removing 8 items Cronbach's *α* coefficient for the PAS of 20 items in Spanish context was 0.84 which was still in good range.

The current study has several limitations that must be acknowledged. First, the samples were recruited from a nonclinical sample, one should be cautious while generalizing this finding in a clinical setting. Second, the current study test−retest reliability and divergent validity were not examined in this study, and this should be the focus of future research. Third, limitation of this study was based solely on the mothers' report for factor analysis. Fourth, this study used on mother only. But other both informants (father and mothers), teachers and other assessment methods (such as direct behavioral observation) was not used.

## 5 CONCLUSION

The Nepalese version of the PAS demonstrated to have good psychometric properties in a sample of three to 6‐year‐old children. Age and sex differences across PAS scales were found to be statistically significant in mother's reports. Preliminary exploratory factor analyses results suggested that the items of the PAS‐ Nepali version do not reflect the five categories in anxiety in a clear way. This is particular in separation anxiety, social phobia, and obsessive‐compulsion. The Confirmatory factor analysis of PAS‐28 items showed poor fit of five factor original model for the data. However, adjusted five factor original model after removing three items (25‐item version) showed better fit for the data. Cronbach's *α* for the PAS scale was in good range (0.87).

The PAS could be considered as a potential instrument to screen and assess the type and severity of anxiety problems. Moreover, it can also be a good supporting tool for clinicians and researchers, as it is short and easy to administer.

## 6 IMPLICATION OF STUDY

1. In the Nepalese context there is inadequate number of mental health experts such as psychiatrist, clinical psychologist, and psychiatric nurse.
2. Subsequently there is minimal number of specialized clinician in child mental health. This ultimately leads to delayed identification of cases resulting into severity of the mental illness and difficulty in the management. The screening tools could provide identification of most at risk children and provide opportunities for interventions.
3. It is important to identify children who are showing first signs of anxiety problems and therefore at risk of developing anxiety disorders. There is no any validated tool in Nepalese context. So, PAS tool could be helpful for community lay person in primary health care for case identification and referral. So, we can provide timely management of case.
4. PAS is short, easily accessible and easy to administer.
5. The present study finds support for the psychometric properties of the Nepali translation of PAS‐Parent version for three to 6 years of children. Result from factor analysis suggest that items on Nepali version of PAS reflect five categories of anxiety. Hence, the study supports the utility of PAS‐Parent Version of 25 items which can be used for screening anxiety symptoms in children.
6. It can be used in research and clinical work.
7. We can conduct this study on PAS for children through Online Photo voice. This is one of the most recent and effective innovative qualitative research methods.^32^, ^33^, ^34^

## AUTHOR CONTRIBUTIONS

**Sabina Maharjan**: Conceptualization; data curation; formal analysis; funding acquisition; investigation; methodology; project administration; resources; software; writing—original draft; writing—review and editing. **Mita Rana**: Conceptualization; supervision; validation; visualization; writing—original draft; writing—review and editing. **Bidusha Neupane**: Conceptualization; supervision; visualization. **Sujan Rijal**: Conceptualization; visualization; writing—review and editing. **Suraj Shakya**: Conceptualization; methodology; project administration; supervision; validation; visualization; writing—original draft; writing—review and editing. **Pramesh Man Pradhan**: Conceptualization; visualization; writing—original draft. **Saroj Prasad Ojha**: Supervision; validation. **Kamal Gautam**: Supervision; validation; visualization; writing—review and editing. **Rakesh Singh**: Conceptualization; supervision; validation; visualization; writing—original draft; writing—review and editing.

## CONFLICT OF INTEREST

The authors declare no conflicts of interest.

## TRANSPARENCY STATEMENT

The lead author Sabina Maharjan affirms that this manuscript is an honest, accurate, and transparent account of the study being reported; that no important aspects of the study have been omitted; and that any discrepancies from the study as planned have been explained.

## Supporting information

Supporting information.

Supporting information.

## Data Availability

The data that support the findings of this study are available from the corresponding author upon reasonable request.
