# Supplementary material for: Psychometric properties of Nepalese preschool anxiety scale among preschool children: A cross‐sectional study
Source: Health Sci Rep. 2022 Sep 3;5(5):e808. doi: 10.1002/hsr2.808 (PMC9440761; doi:10.1002/hsr2.808)
Supplement: Supplementary file 1 — Supporting information. [file HSR2-5-e808-s002.pdf]

Preschool Anxiety Scale (Parent Report)

बलबालिकाको चिन्ताको मापन

(अभिभावकको रिपोर्ट)

तपाईंको नामः .... मितिः ....

तपाईंको बच्चाको नामः ....

तल बालबालिका लाई व्याख्या गर्ने बुदाहरु उल्लेख गरिएको छ । हरेक जानकारीको लागि तपाईंको बच्चालाई मिल्ने प्रतिक्रियामा गोलो चिन्ह लगाउनुहोस् । यदि त्यो जानकारी धेरै जसो लागु हुन्छ भने कृपया **४** मा, प्रायःजसो लागु हुन्छ भने कृपया **३** मा, कहिलेकाहीँ लागु हुन्छ भने कृपया **२** मा र खासै लागु हुँदैन भने कृपया १ मा र यदि त्यो पटक्कै लागु हुँदैन भने कृपया **०** मागोलो चिन्ह लगाउनुहोस् ।

		पटक्कै लागु हुँदैन	खासै लागु हुँदैन	कहिलेकाही लागु हुन्छे	प्रायःजसो लागु हुन्छ	धेरै जसो लागु हुन्छ
१	चिन्ता रोक्न गाह्रो हुने	०	१	२	३	४
२	अरुको अगाडि मुर्खता पूर्वक केहि काम गर्छु कि भने चिन्ता लाग्ने	०	१	२	३	४
३	आफुले गरेका काम ठिक छ कि छैन भनी निरन्तर जाँच गरिरहने (उदाहरणको लागि ढोका लगायो कि लगाएन, धारा बन्द गऱ्यो कि गरेन)	०	१	२	३	४
४	चिन्ताले गर्दा तनाव, छटपटी वा छिटै चिड्चिडानि हो	०	१	२	३	४
५	आफु भन्दा ठूलो व्यक्तिलाई सहायता माग्न डराउने (उदाहरणको लागि - शिक्षकहरु)	०	१	२	३	४
६	तपाई (अभिभावक) विना घर देखि बाहिर वा सुत्न हिचकिचाउने	०	१	२	३	४
७	उचाई देखि डराउने (अग्लो ठाउँहरु)	०	१	२	३	४
८	चिन्ताले गर्दा सुत्न हुने	०	१	२	३	४
९	दिनभरीमा घरी घरी हात धोई रहने	०	१	२	३	४
१०	भीड भएको र साँगुरो ठाउँहरु देखि डराउने	०	१	२	३	४
११	नचिनेको व्यक्तिसँग भेट्न वाबोल्न डराउने	०	१	२	३	४
१२	आफ्नो आमाबुवालाई केही नराम्रो हुन्छ भनेर चिन्तागर्नु	०	१	२	३	४
१३	आधिबेरी देखी डराउने	०	१	२	३	४

१४	दिनभरिमा धरै समय धेरै कुराहरुमा चिन्ता लिएर बिताउने	०	१	२	३	४
१५	कक्षाको अगाडि बोल्न डराउने (पूर्व विद्यालयीय) उदाहरणको लागिः हेरेर बोल्नु	०	१	२	३	४
१६	उसलाई नराम्रो घटना परिन्छ (उदाहरणको लागि हराउनु वा अपहरणमा पर्नु) र त्यस्को कारण अभिभावक सँग छरिनु पर्ला भन्ने चिन्ता लाग्ने	०	१	२	३	४
१७	पौडी खेल्न डराउनु	०	१	२	३	४
१८	कुनै नराम्रो घटना हुनबाट रोक्न ठ्याक्क त्यस्तै किसिमले सामानहरु मिलाएर राख्नुपर्ने ।	०	१	२	३	४
१९	अरु मानिसहरुको अगाडि केहि लजित काम गर्छु भनेर चिन्ता हुने ।	०	१	२	३	४
२०	किरा वा माकुरासँग डराउने ।	०	१	२	३	४
२१	नराम्रो वा वाहियात लाग्ने सोचबिचार वा चित्रहरु बरम्बार आइलाग्ने ,	०	१	२	३	४
२२	तपाइले उसलाई विद्यालयहरु व अरु कोहिसँग छोडेर जाँदा चिन्तित हुने	०	१	२	३	४
२३	अरु बच्चाहरुको समुहमा जान र उनीहरुको क्रियाकलापमा संलग्न हुन डराउने	०	१	२	३	४
२४	कुकुर देखि डराउने	०	१	२	३	४
२५	तपाई अभिभावकबाट टाढा भएको नराम्रो सपनाहरु देख्ने ।	०	१	२	३	४
२६	अध्याँरो देखी डराउने	०	१	२	३	४
२७	कुनै नराम्रो घटना हुनबाट रोक्न मनमा विशेष बिचारहरु (उदाहरणको लागि केहि)	०	१	२	३	४
२८	आवश्यक नभएको बेलामा पनि अरुको आश्वासन खोज्ने	०	१	२	३	४
२९	तपाईंको बच्चाले के कुनै एकदम नराम्रो वा आघातपूर्ण घटनाको अनुभव गरेको छ ? (उदाहरणको लागिःगम्भीर दुर्घटना, पारिवारीक सदस्य/साथीको निधन, दुर्व्यवहार, लुटपाट, प्रकोप)	हो			होइन	

तपाईंको बच्चाले अनुभव गरेको घटनालाई छोटकरीमा व्याख्या गर्नुहोस्: ____________________

____

____

____

____

यदि तपाईंले प्रश्न २९मा होइन मा चिह्न लगाउनुभएको हा भने, प्रश्न

३० - ३४ सम्मको जवाफ दिनुपर्दैन। यदिहो मा चिह्न लगाउनुभएको छ भने, तलका प्रश्नहरुको जवाफ दिनुहोस्।

के तलका वाक्यहरुले त्यो घटना देखीको तपाईंको बच्चाको व्यवहारलाई वर्णन गर्दछ ?

३०	त्यो घटनाको बारेमा नराम्रो वा डरलाग्दो सपनाहरु देख्ने	०	१	२	३	४
३१	त्यो घटना सम्भिन्छ अनि चिन्तित हुने	०	१	२	३	४
३२	त्यो घटना सम्भाइदिदा चिन्तित हुने	०	१	२	३	४
३३	फेरी त्यो घटना वा सामना गरेको जस्तो व्यवहार गर्ने	०	१	२	३	४
३४	त्यो घटना सम्भाइदिदा डरको शारिरीक लक्ष्यणहरु देखाउने (उदाहरणको लागि पसिना आउने, हात काम्ने वा मुटु हान्ने)	०	१	२	३	४
