# Supplementary material for: Psychometric properties of Nepalese preschool anxiety scale among preschool children: A cross‐sectional study
Source: Health Sci Rep. 2022 Sep 3;5(5):e808. doi: 10.1002/hsr2.808 (PMC9440761; doi:10.1002/hsr2.808)
Supplement: Supplementary file 2 — Supporting information. [file HSR2-5-e808-s001.pdf]

PRESCHOOL ANXIETY SCALE
(Parent Report)

Your Name: ______________________ **Date:** ___________________

Your Child's Name: ______________________

Below is a list of items that describe children. For each item please circle the response that best describes your child. Please circle the **4** if the item is **very often true, 3** if the item is **quite often true, 2** if the item is **sometimes true, 1** if the item is **seldom true** or if it is **not true at all** circle the **0**. Please answer all the items as well as you can, even if some do not seem to apply to your child.

		Not True at All	Seldom True	Sometimes True	Quite Often True	Very Often True
1	Has difficulty stopping him/herself from worrying…………………	0	1	2	3	4
2	Worries that he/she will do something to look stupid in front of other people…………………………………………………………	0	1	2	3	4
3	Keeps checking that he/she has done things right (e.g., that he/she closed a door, turned off a tap)………………..	0	1	2	3	4
4	Is tense, restless or irritable due to worrying………………………	0	1	2	3	4
5	Is scared to ask an adult for help (e.g., a preschool or school teacher)………………………………………………………………...	0	1	2	3	4
6	Is reluctant to go to sleep without you or to sleep away from home………………………………………………………………..	0	1	2	3	4
7	Is scared of heights (high places)……………………………………	0	1	2	3	4
8	Has trouble sleeping due to worrying………………………………	0	1	2	3	4
9	Washes his/her hands over and over many times each day…….	0	1	2	3	4
10	Is afraid of crowded or closed-in places……………………………..	0	1	2	3	4
11	Is afraid of meeting or talking to unfamiliar people…………………	0	1	2	3	4
12	Worries that something bad will happen to his/her parents………..	0	1	2	3	4
13	Is scared of thunder storms…………………………………………..	0	1	2	3	4
14	Spends a large part of each day worrying about various things…..	0	1	2	3	4
15	Is afraid of talking in front of the class (preschool group) e.g., show and tell…………………………..……………………	0	1	2	3	4
16	Worries that something bad might happen to him/her (e.g., getting lost or kidnapped), so he/she won't be able to see you again………………………………………………………………..	0	1	2	3	4
17	Is nervous of going swimming………………………………………...	0	1	2	3	4

		Not True at All	Seldom True	Sometimes True	Quite Often True	Very Often True
18	Has to have things in exactly the right order or position to stop bad things from happening…………………………………………	0	1	2	3	4
19	Worries that he/she will do something embarrassing in front of other people……………………………………………………………	0	1	2	3	4
20	Is afraid of insects and/or spiders…………………………………	0	1	2	3	4
21	Has bad or silly thoughts or images that keep coming back over and over…………………………………………………………………	0	1	2	3	4
22	Becomes distressed about your leaving him/her at preschool/school or with a babysitter……………………………...	0	1	2	3	4
23	Is afraid to go up to group of children and join their activities……..	0	1	2	3	4
24	Is frightened of dogs……………………………………………………	0	1	2	3	4
25	Has nightmares about being apart from you……………………...	0	1	2	3	4
26	Is afraid of the dark……………………………………………………	0	1	2	3	4
27	Has to keep thinking special thoughts (e.g., numbers or words) to stop bad things from happening………………………………………	0	1	2	3	4
28	Asks for reassurance when it doesn't seem necessary…………..	0	1	2	3	4
29	Has **your child ever experienced anything really bad or traumatic** (e.g., severe accident, death of a family member/friend, assault, robbery, disaster) …………………………	**YES**	**NO**			

Please briefly describe the event that your child experienced……

If you answered **NO** to **question 29,** please **do not** answer questions 30-34**. If you answered YES, please DO** answer the following questions**.**

Do the following statements describe your child's behaviour since the event?

		Not True at All	Seldom True	Sometimes True	Quite Often True	Very Often True
30	Has bad dreams or nightmares about the event……………………	0	1	2	3	4
31	Remembers the event and becomes distressed……………………	0	1	2	3	4
32	Becomes distressed when reminded of the event…………………	0	1	2	3	4
33	Suddenly behaves as if he/she is reliving the bad experience……	0	1	2	3	4
34	Shows bodily signs of fear (e.g., sweating, shaking or racing heart) when reminded of the event …………………………………	0	1	2	3	4

© 1999 Susan H. Spence and Ronald Rapee
